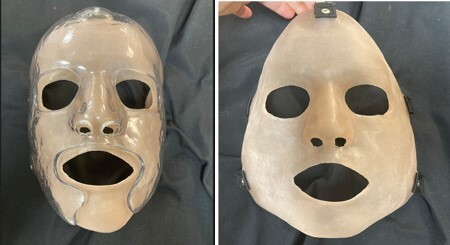# 594 A Novel Sequential Compression Appliance to Reduce Scarring of the Face

**DOI:** 10.1093/jbcr/irae036.228

**Published:** 2024-04-17

**Authors:** Michelle N Dwertman, Henry B Huson

**Affiliations:** University of Cincinnati Burn Unit, Cincinnati, OH; LSUHSC New Orleans, New Orleans, Louisiana; University of Cincinnati Burn Unit, Cincinnati, OH; LSUHSC New Orleans, New Orleans, Louisiana

## Abstract

**Introduction:**

A transparent facial orthosis (TFO) is crucial for managing scars in burn cases. However, TFOs are typically static and rigid, lacking adaptability to facial movements. Their effectiveness depends on the ability to conform to each patient's unique facial contours and applying the correct amount of pressure to scars. Crafting or modifying TFOs is a time-consuming, skilled, and costly process. To address these challenges, we've developed the flexible and adaptable Sequential Compression Appliance to Reduce Scarring (SCARS) mask in collaboration with a local prosthetic and orthotic lab. This innovation is seamless, allows natural facial movement, and can be easily customized by a trained burn therapist, providing effective scar management.

**Methods:**

SCARS Mask Design: The SCARS mask consists of two key components: a rigid transparent outer layer akin to traditional TFOs and a pliable High Consistency Rubber (HCR) silicone layer. These can be used together or separately. The inner layer offers a minimum 20mmHg pressure displayed by the Kikuhime device and remains flexible to accommodate natural facial movements, including temporomandibular joint (TMJ) mobility. The silicone layer is elastic, able to conform to facial contours, and can be color-tinted to individual skin tones. The mask features multidirectional embedded harness attachments, is lighter in weight than traditional orthotics, and can be trimmed with scissors.

Process: To create the SCARS mask, FDA-approved Platinum Silicone HCR is merged, pigment tinted, and rolled to the desired thickness. The HCR silicone is heated and molded based on a 3D scan of their face.

Clinical Trials: Successful trials on three patients with severe facial burns who exhibit significant scarring and microstomia progression, provided valuable insights. The SCARS mask's performance was noted, confirming its effectiveness.

Measurement: Each individual was given Likert scale to rate their experience.

**Results:**

In the trials involving three patients with severe facial burns:

Patient Preference: All patients unanimously preferred the SCARS mask over traditional TFOs, rating it five on the Likert scale.

Improved Eating and Mandibular Movement: Patients experienced significant improvements in eating comfort and mandibular movement while using the SCARS mask, with an average Likert scale rating of five.

**Conclusions:**

The SCARS mask represents a significant advancement in the field of facial orthosis. Its innovative design, flexibility, adaptability, and customizable features offer a promising solution for individuals with burn-related facial scarring, enhancing both comfort and treatment effectiveness.

**Applicability of Research to Practice:**

This pliable appliance enhances comfort and adherence in managing facial scar hypertrophy. Its customization, allowing color-tinting to match skin tones, and cost-effective modification make it a valuable tool for improving scar management outcomes of facial scarring.